# Comparison of Immune Indicators Related to Phagocytosis of Five Species of Sea Urchins under Artificial Infection with the Pathogenic Bacterium of Black Mouth Disease

**DOI:** 10.3390/biology13070495

**Published:** 2024-07-03

**Authors:** Wenzhuo Tian, Zhong Wang, Xiaofei Leng, Peng Liu, Hao Guo, Xuechun Jiang, Fanjiang Ou, Tongshan Jia, Jun Ding, Weijie Zhang, Yaqing Chang

**Affiliations:** 1 Key Laboratory of Mariculture & Stock Enhancement in North China’s Sea, Ministry of Agriculture and Rural Affairs, Dalian Ocean University, Dalian 116023, China; 15120757859@163.com (W.T.); wangzhong950319@163.com (Z.W.);; 2 Dalian Haibao Fisheries Limited, Dalian 116041, China; 3 National Engineering Research Laboratory of Marine Biotechnology and Engineering, Ningbo University, Ningbo 315211, China; 4 Key Laboratory of Aquacultural Biotechnology (Ningbo University), Ministry of Education, Ningbo 315211, China; 5 Collaborative Innovation Center for Zhejiang Marine High-Efficiency and Healthy Aquaculture, Ningbo University, Ningbo 315211, China; 6 Key Laboratory of Marine Biotechnology of Zhejiang Province, Ningbo University, Ningbo 315211, China

**Keywords:** disease-resistant, coelomocytes, phagocytosis, immune indicator

## Abstract

**Simple Summary:**

Analyzing the immune mechanism of sea urchins against pathogens is the basis of disease-resistant breeding. When faced with pathogen invasion, the phagocytosis of sea urchin coelomocytes plays a major immune role. However, it is unclear how can phagocytosis-related immune indices change can help sea urchins resist disease. To answer this question, this study compared the differences in immune indices related to phagocytosis between susceptible and disease-resistant sea urchins after pathogen infection. We found that the apoptosis and necrosis rate of phagocytes, phagocytic index, ACP, ROS and T-AOC may be used as indicators of disease resistance in sea urchins. Disease resistance standards in immune indices can be summarized as phagocytosis increases greatly in the early infection stage and decreases timely to a normal level after killing the pathogen in a short period. The results of this paper can provide important references for disease-resistant breeding of sea urchins.

**Abstract:**

To screen for immune indicators closely related to disease resistance, two species of sea urchin susceptible to black mouth disease (*Strongylocentrotus intermedius*, *S. intermedius* ♀ × *Heliocidaris crassispina* ♂) and three species of sea urchin resistant to black mouth disease (*H. crassispina*, *H. crassispina* ♀ × *S. intermedius* ♂ and *Mesocentrotus nudus*) were artificially infected with the black mouth pathogen *Vibrio echinoideorum*. The phagocytosis-related immune indices of the five sea urchin species were compared at different time points post-infection. The results demonstrated that the parameters such as apoptotic rate of phagocytes, mean contribution value (MCV) of single effective phagocyte on Acid Phosphatase (ACP), Reactive Oxygen Species (ROS), and Total Antioxidant Capacity (T-AOC) of the five sea urchin species first increased and then decreased after infection. The key time points were 3 h to 6 h and 48 h post-infection when the black mouth disease-resistant and susceptible sea urchins demonstrated differences. At 3 h or 6 h post-infection, the up-regulation folds in MCV of ACP, ROS and T-AOC of black mouth disease-resistant sea urchins were considerably higher than that of the susceptible sea urchins. At 6 h post-infection, the apoptosis rate and the phagocytic index (PI) of the black mouth disease-resistant sea urchins were significantly higher than those of the susceptible sea urchins (*p* < 0.05). At 48 h post-infection, the necrosis rate of phagocytes, MCV of ACP and MCV of ROS of the black mouth disease-resistant sea urchins were significantly lower than those of the susceptible sea urchins (*p* < 0.05). The apoptosis and necrosis rate of phagocytes, PI, and MCV on ACP, ROS may be used as indicators of disease resistance in sea urchins. Disease resistance standards in immune indices can be summarized as phagocytosis increases greatly in the early infection stage and decreases timely to a normal level after killing the pathogen in a short period.

## 1. Introduction

Edible sea urchins, apart from their good flavor, are also nutritious and have high medical and healthcare functions [1]. Several regions in the world traditionally eat sea urchins. Research on artificial culture and seedling production has therefore been gradually carried out to meet the human demand for sea urchins [2,3,4,5]. In China, Japan, and other countries, *Strongylocentrotus intermedius* is the primary species of cultured sea urchin. In recent years, various bacterial diseases such as black mouth disease, spotting disease, and lesion syndrome have affected sea urchin culture [5,6,7,8,9,10,11,12,13,14,15,16], demonstrating a growing trend. Diseases in sea urchins are primarily bacterial and related to water temperature [7,9,10,11]. Since the culture is limited to the sea-based method, it is not possible to control diseases by monitoring water temperature and the number of pathogenic bacteria. Breeding for disease-resistant varieties of sea urchins should therefore be an effective way to prevent and control the large-scale death of cultured sea urchins and improve the efficiency of sea urchin culture.

Analyzing the immune mechanism of sea urchins against pathogens is the basis of disease-resistant breeding. Invertebrates have a primitive immune response to diseases, lack real immune antibodies, and can only rely on their innate nonspecific immune system to resist the infection of foreign pathogens [17]. The immune system of echinoderms is primarily composed of phagocytes, humoral complement components, and transcription factors induced by various pathogens. For example, sea cucumbers can use phagocytes in the coelomic fluid to phagocytize foreign substances, thereby maintaining body health [18,19]. Phagocytes constitute the first line of defense against foreign pathogens in sea urchins, which ingest and destroy foreign substances by phagocytosis [20,21,22]. Studies have demonstrated that when sea urchins are affected by external factors, phagocytosis will change accordingly, and modulator-like molecules and complement C3 can promote phagocytosis [23,24,25]. When affected by the disease, the expression of phagocytosis-related immune pathway genes such as *C3-pre*, *Caspase-8* and *Clec4g* of sea urchin decreased, resulting in a decrease in phagocytosis [14,26]. Black mouth disease is one of the most threatening diseases to the sea urchin *S. intermedius*, and it usually erupts during the low-temperature period between spring and summer. The typical symptoms of diseased sea urchins are necrosis of the muscle tissue in the teeth, blackening of the perioral membrane, and an inability to engage in normal attachment and feeding activities. The pathogen of *S. intermedius* black mouth disease has been identified as *Vibrio echnoideorum*, infected with which *S. intermedius* usually die after 2 days [27]. Our previous study [16] reported the change pattern of phagocytosis of the susceptible *S. intermedius* when infected by the pathogen of black mouth disease. It was observed that *S. intermedius* enhanced phagocytosis by increasing the apoptosis rate of phagocytic amoebocytes, acid phosphatase (ACP) activity, reactive oxygen species (ROS) content, and total antioxidant capacity (T-AOC) in the early stage of infection. Phagocytosis decreased considerably in the later stage of infection. However, it is unclear how can phagocytosis-related immune indices change can help sea urchins resist disease. The most direct way to answer this question would be to compare the performance differences of these immune indices of susceptible and disease-resistant sea urchins after pathogen infection. Disease resistance standards in immune indices may be set as per these differences and used in selective breeding for disease resistance.

In aquaculture practice, *Mesocentrotus nudus*, *Heliocidaris crassispina*, and *H. crassispina* ♀ × *S. intermedius* ♂ were found to have strong resistance to black mouth disease, while *S. intermedius*, *S. intermedius* ♀ × *H. crassispina* ♂ were found to be extremely susceptible to the disease. This study intends to determine the standard for disease resistance by comparing the differences in phagocytosis-related parameters of the five sea urchins infected by the pathogen of black mouth disease to provide a technical reference for the breeding for disease resistance in sea urchins.

## 2. Material and Methods

### 2.1. Materials

Five sea urchin species, i.e., *S. intermedius*, *S. intermedius* ♀ × *H. crassispina* ♂, *H. crassispina*, *H. crassispina* ♀ × *S. intermedius* ♂ and *M. nudus*, were artificially bred in September 2021 at the Key Laboratory of Mariculture & Stock Enhancement in North China’s Sea, Ministry of Agriculture and Rural Affairs, Dalian, China. Prior to the experiment, these five species of sea urchins were carefully cultured to avoid natural infections of any disease. At 14 months of age, 69 individuals of each sea urchin species (diameters of *S. intermedius*, *S. intermedius* ♀ × *H. crassispina* ♂, *H. crassispina*, *H. crassispina* ♀ × *S. intermedius* ♂ and *M. nudus* were approximately 2 cm, 4 cm, 2 cm, 2 cm, and 3 cm, respectively) were randomly selected as experimental animals. The pathogenic bacteria of black mouth disease were isolated from naturally diseased *S. intermedius* and were identified as *Vibrio echinoideorum* [27].

### 2.2. Method

#### 2.2.1. Experimental Design

Five species of sea urchins were randomly placed in 60 aliquots in three 20 L sterilized water tanks, and the experiment was started after three days of temporary incubation. The experiment was conducted under the conditions of the mean water temperature of 15.8 °C, salinity of 31.1, pH of 8.1, and dissolved oxygen of 8.1 mg/L. A 1 mL sterile syringe needle was used to pierce the body cavity of the sea urchin from the perioral membrane to damage the sea urchin (a preliminary experiment was conducted, and the results indicated that this wound will heal within 36 h and sea urchins will not die from this wound). Subsequently, 10 μL of the pathogenic bacteria of black mouth disease *V. echinoideorum* was added to the water tank at 10^8^ cfu/mL concentration to obtain a 10^2^ cfu/mL initial concentration of the soaking infection. Samples were collected before infection (0 h) and 1 h, 3 h, 6 h, and 48 h after infection using the following methods: Five sea urchins were randomly selected from each tank, and each was dissected and 1 mL of coelomic fluid was retrieved. After mixing, 100 μL was retrieved for cell classification and counting. Another 900 μL was retrieved for detecting apoptosis and necrosis rates of phagocytes. The remaining coelomic fluid was sub-packed into six 1.5 mL centrifuge tubes and centrifuged at 4 °C for 15 min at 3000× *g*. The supernatant was discarded, and the precipitated coelomocytes were stored at −80 °C for subsequent determination of phagocytosis-related immune parameters. The same sampling method was applied three times at each time point as three replications.

Nine of each remaining sea urchin species were placed into five 20 L sterilized sinks, and the experiment was started after 3 days. Subsequently, 10 μL of monodisperse green fluorescent microspheres (BaseLine, Tianjin, China; 1.0 μm) were injected into the cavity of the sea urchin body from the perioral membrane at a concentration of 10 mg/mL using a 1 mL sterile syringe. Samples were retrieved at 3 h, 6 h, and 48 h post-injection. For the sampling method, three sea urchins were randomly sampled from each sink, and 100 μL of coelomic fluid was retrieved for the detection of phagocytic rate and phagocytic index post-infection.

#### 2.2.2. Categorical Counts of Coelomocytes

Coelomocytes were counted using a hemocytometer with reference to the classification method by Smith et al. [28].

#### 2.2.3. Determination of Phagocytosis-Related Immune Parameters

ACP activity, ROS content, T-AOC, as well as immune parameters, including apoptosis and necrosis rate of phagocytes, were measured using a kit (Shanghai Biyuntian Biotechnology Co., Ltd., Shanghai, China). The assay steps were strictly adhered to according to the instructions on the kit. The apoptotic rate of phagocytes was calculated as the number of apoptotic cells divided by the total number of phagocytes within each field under a fluorescence microscope (3 visual fields were taken for each sample to ensure that the number of observed cells exceeded 100). The necrotic rate of phagocytes was calculated as the number of necrotic phagocytes divided by the total number of phagocytes. The phagocytic rate was calculated as the number of phagocytes engulfing fluorescent microspheres divided by the total number of phagocytes within each field under a fluorescence microscope (10 visual fields were taken for each sample to ensure that the number of observed cells exceeded 100). The phagocytic index was obtained as the total number of fluorescent microspheres engulfed by phagocytes participating in phagocytosis divided by the total number of phagocytes within each field under a fluorescence microscope (10 visual fields were taken for each sample).

#### 2.2.4. Data Analysis

Mean contribution value (MCV) of a single phagocyte on each immune parameter = measurement of each immune parameter/(number of phagocytes − number of necrotic phagocytes) [16].

Two-way ANOVA was first used to analyze the species-time interaction effect on each immune parameter using SPSS 20.0. Significant species-time interaction effects were detected for almost all the parameters (Table 1). Thus, one-way ANOVA was used to analyze the effect of species on each immune parameter within each time point post-infection and the effect of infection time on each immune parameter within each species. Prior to variance analysis, data were tested for normality using a one-sample Kolmogorov–Smirnov test. Natural logarithmic transformation was used to transform data that did not conform to normality. Levene’s test of equality of error variances were followed by variance analysis. The Tukey multiple comparison method was used to analyze the significance of differences among the five sea urchin species within each time point, and the significance of differences between each time point post-infection and 0 h within each sea urchin species. The significance level was set at *p* < 0.05 and the very significance level was set at *p* < 0.01.

## 3. Results

### 3.1. Effects of Species, Time and Species–Time Interaction on Immune Parameters

The effects of species, time and species–time interaction on immune parameters are listed in Table 1. Both species and time showed significant (*p* < 0.05) or very significant (*p* < 0.01) effects on all the immune parameters. The species–time interaction effects on all the immune parameters except for phagocytic rate were significant (*p* < 0.05) or very significant (*p* < 0.01). Due to the significant species–time interaction effects, subsequent results were based on one-way ANOVA.

### 3.2. Comparison of Coelomocytes Density Changes

**Figure 1:**
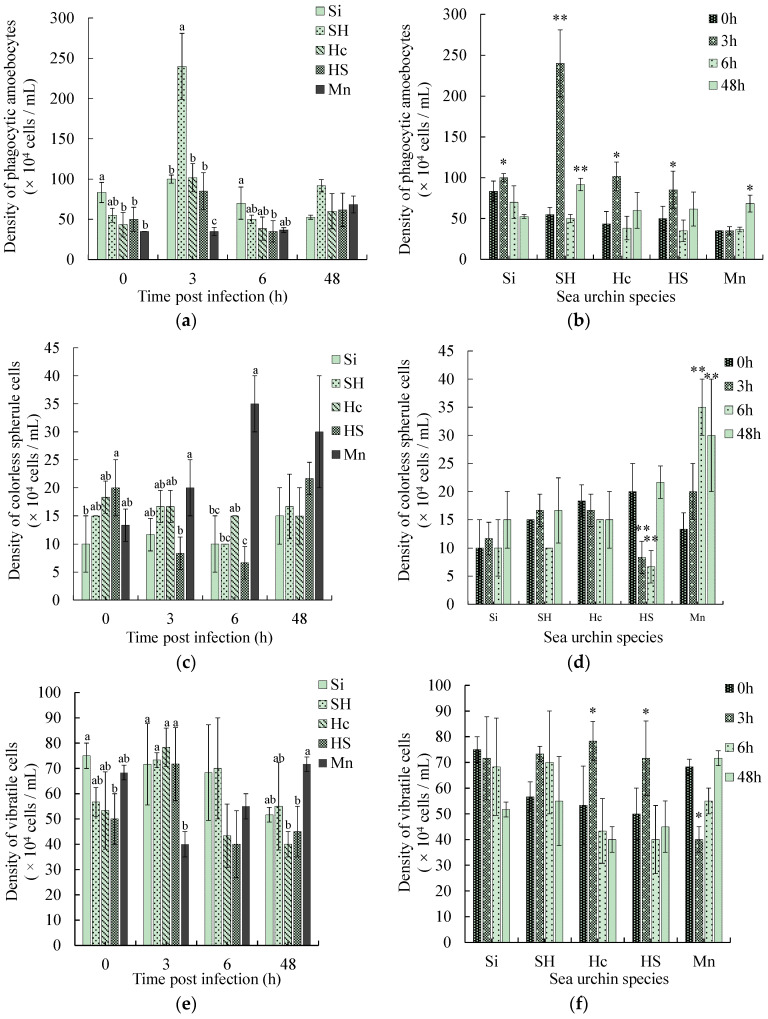

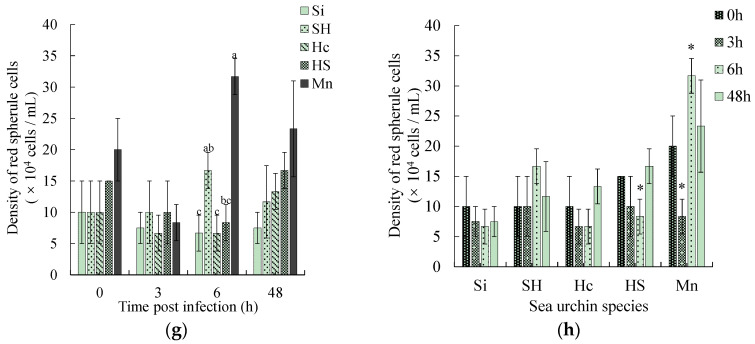
Density changes of coelomocytes in five species of sea urchins post-infection. Note: Si: *S. intermedius*, SH: *S. intermedius* ♀ × *H. crassispina* ♂, Hc: *H. crassispina*, HS: *H. crassispina* ♀ × *S. intermedius* ♂. (**a**,**c**,**e**,**g**) are the comparisons among five sea urchin species within a time point, in which different superscript lowercase letters indicate significant differences among sea urchin species within each time point. (**b**,**d**,**f**,**h**) are the comparisons among time points within species, in which ** indicates a significant difference from 0 h within each species (*p* < 0.01), * indicates a significant difference from 0 h within each species (*p* < 0.05).

As presented in Figure 1, at 0 h of infection, the density of phagocytic amoebocytes in the coelomic fluid of *S. intermedius* was (83.33 ± 12.58) × 10^4^ cells/mL, which was significantly higher (*p* < 0.05) than that in the three black mouth disease-resistant sea urchins (Figure 1a). The density of colorless spherule cells in the coelomic fluid of the black mouth disease-resistant sea urchins (*H. crassispina* ♀ × *S. intermedius* ♂) was (20.00 ± 13.33) × 10^4^ cells/mL, which was significantly higher than that of the sea urchins susceptible to black mouth disease (*S. intermedius*) [(10.00 ± 5.00) × 10^4^ cells/mL] (*p* < 0.05) (Figure 1c). The density of vibratile cells in the coelomic fluid of the susceptible sea urchins *S. intermedius* was the highest among the five species, reaching (75.00 ± 5.00) × 10^4^ cells/mL, significantly higher than that of black mouth disease-resistant sea urchins (*H. crassispina* ♀ × *S. intermedius* ♂) (*p* < 0.05) (Figure 1e). After 3 h of infection, the phagocytic amoebocyte densities in two of both susceptible sea urchins (*S. intermedius* and *S. intermedius* ♀ × *H. crassispina* ♂) and disease-resistant sea urchins (*H. crassispina* and *H. crassispina* ♀ × *S. intermedius* ♂) rose to the highest values of (100.00 ± 5.00) × 10^4^ cells/mL, (240.00 ± 40.93) × 10^4^ cells/mL, (101.67 ± 17.56) × 10^4^ cells/mL and (85.00 ± 22.91) × 10^4^ cells/mL, respectively, all significantly higher than before the infection (*p* < 0.05) (Figure 1b). The phagocyte density of sea urchins susceptible to black mouth disease (*S. intermedius* ♀ × *H. crassispina* ♂) was significantly higher than that of the three black mouth disease-resistant sea urchins (*p* < 0.05) (Figure 1a). The vibratile cell density of the two susceptible sea urchins was significantly higher than that of the black mouth disease-resistant sea urchins *M. nudus* (*p* < 0.05) (Figure 1e). At 6 h post-infection, the cell density of the colorless spherule of black mouth disease-resistant sea urchin *M. nudus* increased highly significantly (*p* < 0.01) compared to before infection (Figure 1d) and was significantly higher (*p* < 0.05) than that of the two sea urchins susceptible to black mouth disease (Figure 1c). The density of red spherule cells of *M. nudus* increased significantly (*p* < 0.05) at 6 h and reached a maximum value of (31.67 ± 2.89) × 10^4^ cells/mL (Figure 1h), significantly higher (*p* < 0.05) than that of the susceptible sea urchin *S. intermedius* (Figure 1g). At the late stage of infection (48 h), there was no significant difference in phagocytosis, colorless spherule cells, and red spherule cells among the five sea urchins (Figure 1a,c,g).

### 3.3. Comparison of Apoptosis Rate and Necrosis Rate of Phagocytes

**Figure 2:**
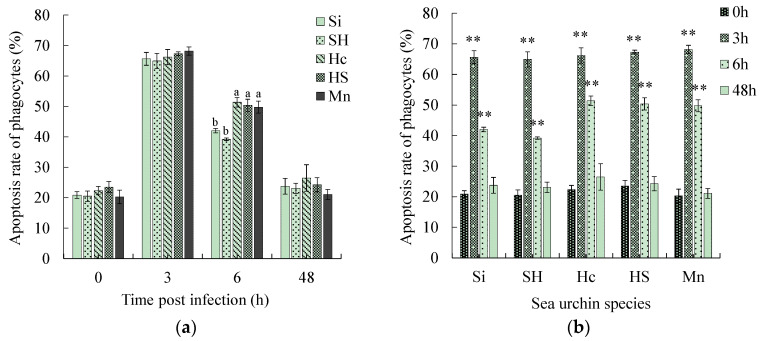

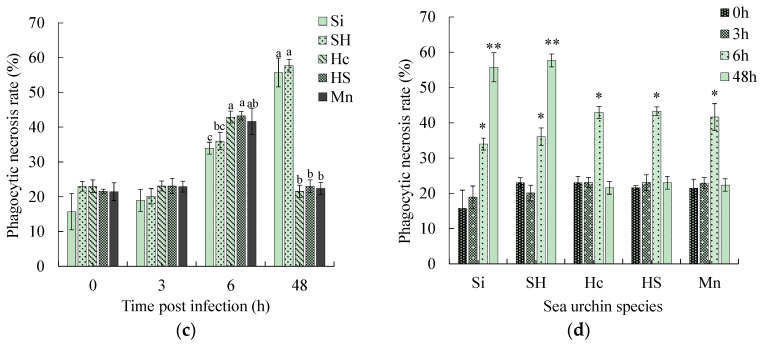
Changes in apoptosis and necrosis rates of phagocytes in five species of sea urchins with infection time. Note: (**a**,**c**) are the comparisons among five sea urchin species within a time point, in which different superscript lowercase letters indicate significant differences among sea urchin species within each time point. (**b**,**d**) are the comparisons among time points within species, in which ** indicates a significant difference from 0 h within each species (*p* < 0.01), * indicates a significant difference from 0 h within each species (*p* < 0.05).

The apoptosis rate of the phagocytes of all five sea urchins reached the highest value 3 h after infection, demonstrating values of (65.63 ± 2.12)%, (64.91 ± 2.48)%, (66.19 ± 2.49)%, (67.30 ± 0.62)%, and (68.19 ± 1.34)% (Figure 2a). After 6 h of infection, the apoptotic rate of phagocytes decreased in both the black mouth disease-resistant and susceptible sea urchins (Figure 2a). However, the apoptotic rate of phagocytes was significantly higher in the three black-mouth disease-resistant sea urchins than in the susceptible ones (*p* < 0.05) (Figure 2a). At 48 h post-infection, the apoptosis rates of all five sea urchins decreased to a level not significantly different from that at 0 h (Figure 2b).

No significant change was observed in the phagocytic necrosis rate of all five sea urchin species at 3 h post-infection (Figure 2c). At 6 h post-stress, the phagocytic necrosis rate of all five sea urchins was significantly higher than that before stress (*p* < 0.05) (Figure 2d). The phagocytic necrosis rate of the two black mouth disease-resistant sea urchins (*H. crassispina* and *H. crassispina* ♀ × *S. intermedius* ♂) was significantly higher than that of the two susceptible sea urchins (*p* < 0.05) (Figure 2c). At 48 h post-infection, the necrosis rate of the susceptible sea urchins increased to the highest values [(55.72 ± 4.10)% and (57.67 ± 1.82)%], while the necrosis rate of the three black mouth disease-resistant sea urchins decreased to a level not significantly different from 0 h (Figure 2d).

### 3.4. Comparison of the Phagocytic Rate and Phagocytic Index

**Figure 3:**
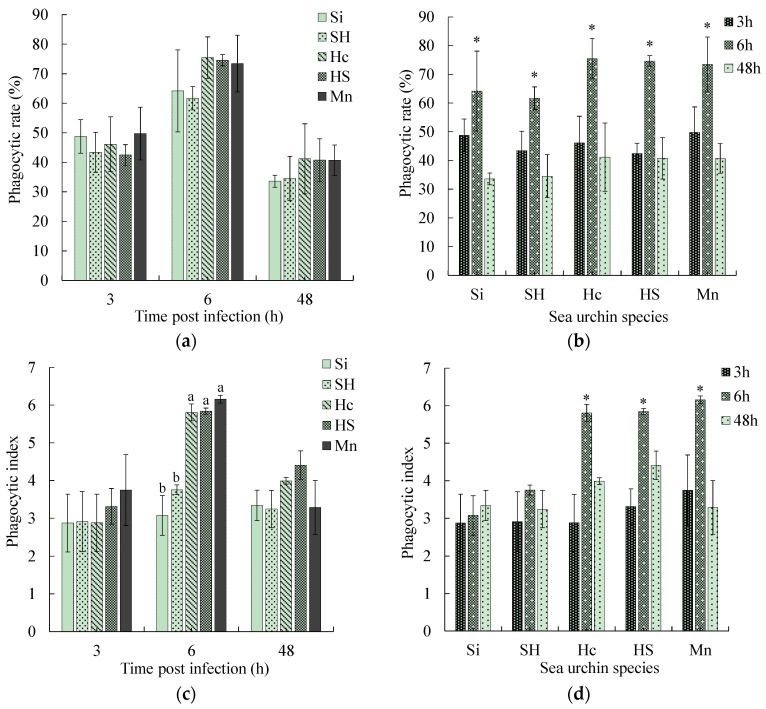
Changes in the phagocytic rate and phagocytic index of five sea urchin species with infection time. (**a**,**c**) are the comparisons among five sea urchin species within a time point, in which different superscript lowercase letters indicate significant differences among sea urchin species within each time point. (**b**,**d**) are the comparisons among time points within species, in which * indicates a significant difference from 0 h within each species (*p* < 0.05).

At 3 h post-infection, the phagocytic rates of the five sea urchin species ranged from 42.46 to 49.73%, with no significant differences among them (Figure 3a). At 6 h post-infection, the phagocytic rates of all five sea urchins increased markedly (*p* < 0.05) (Figure 3b), and the phagocytic rates of the black mouth disease-resistant sea urchins ranged from 73.44 to 75.47%, which were higher than those of the susceptible sea urchins (61.67–64.02%). However, the differences did not reach a significant level (Figure 3a). At 48 h of duress, the phagocytic rates of all five sea urchins decreased significantly (*p* < 0.05) (Figure 3b), and the phagocytic rates of the black mouth disease-resistant sea urchins were still slightly higher than that of the sea urchins susceptible to black mouth disease (Figure 3a).

At 3 h post-infection, the phagocytic indexes of the black mouth disease-resistant sea urchins ranged from 2.88 to 3.75, which was higher than that of the susceptible sea urchins, where the values ranged from 2.87 to 2.91. The difference, however, did not reach a significant level (Figure 3c). At 6 h post-stress, the phagocytic indexes of the black mouth disease-resistant sea urchins increased significantly, reaching 5.81–6.16, which was significantly higher than that of the two sea urchins susceptible to black mouth disease (*p* < 0.05) (Figure 3c). At the late stage of infection (48 h), the phagocytic indexes of the five sea urchins decreased to a level not significantly different from that at 3 h (Figure 3d).

**Figure 4:**
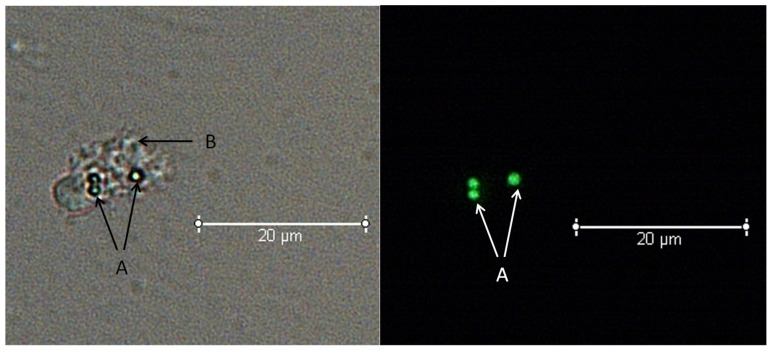
An example of measurement for phagocytic ability. Note: A is a fluorescent microsphere, B is a phagocytic amoebocyte.

An example of measurement for phagocytic ability is shown in Figure 4.

### 3.5. Comparison of Physiological Parameters Related to Phagocytosis

**Figure 5:**
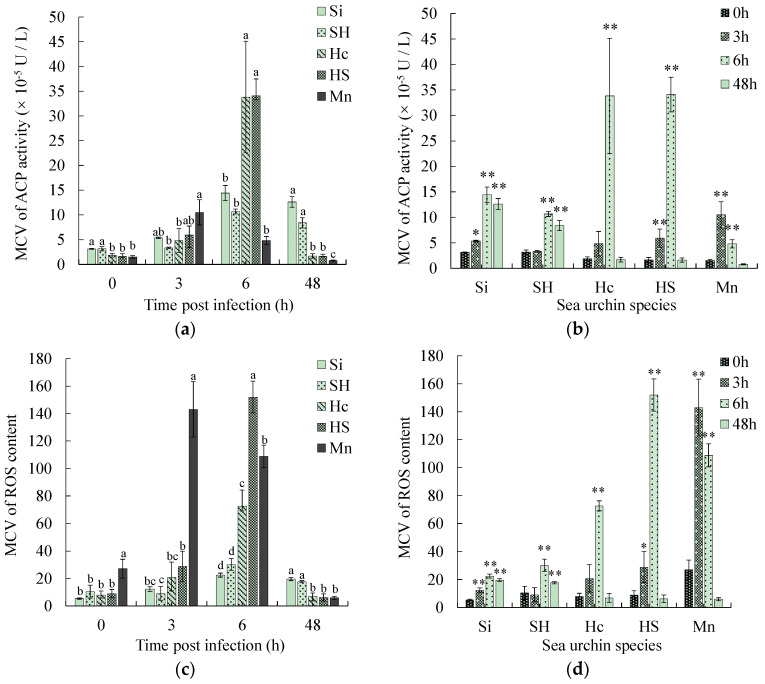

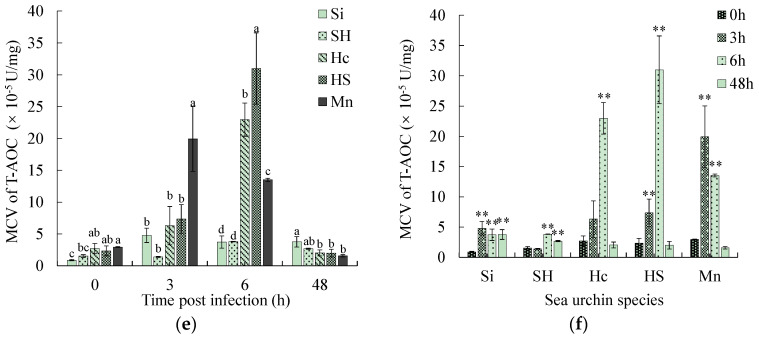
Changes in phagocytosis-related parameters of five species of sea urchins with infection time. (**a**,**c**,**e**) are the comparisons among five sea urchin species within a time point, in which different superscript lowercase letters indicate significant differences among sea urchin species within each time point. (**b**,**d**,**f**) are the comparisons among time points within species, in which ** indicates a significant difference from 0 h within each species (*p* < 0.01), * indicates a significant difference from 0 h within each species (*p* < 0.05).

Phagocytosis-related parameters such as ACP, ROS, and T-AOC of coelomocytes were measured in this study and then calculated as the mean contribution value of single effective phagocytes (MCV). The MCV of the three parameters of ACP, ROS, and T-AOC of the two susceptible sea urchins demonstrated a consistently increasing trend. The three parameters of the three black mouth disease-resistant sea urchins demonstrated a consistent initial increasing and then decreasing trend (Figure 5b,d,f). At 3 h post-infection, the MCV of ACP, ROS and T-AOC of the black mouth disease-resistant sea urchins *M. nudus* was significantly higher than that of the susceptible sea urchins (*p* < 0.05). At 6 h post-infection, the MCV of ACP, ROS and T-AOC of the other two black mouth disease-resistant sea urchins was significantly higher than that of the susceptible sea urchins (*p* < 0.05) (Figure 5a,c,e). The highest up-regulation folds in MCV of ACP ranged from 6.9 to 20.8 in the black mouth disease-resistant sea urchins and from 3.4 to 4.6 in the susceptible ones (Figure 5b). The highest up-regulation folds in MCV of ROS ranged from 5.3–16.9 in the black mouth disease-resistant sea urchins and from 2.9 to 4.1 in the susceptible sea urchins (Figure 5d). The highest up-regulation folds in MCV of T-AOC ranged from 8.5 to 13.2 in the black mouth disease-resistant sea urchins and from 2.5 to 5.4 in the sea urchins susceptible to black-mouth disease (Figure 5f). After 48 h of infection, all three indices of the three black mouth disease-resistant sea urchins decreased to normal levels, while the indices in the susceptible sea urchins remained considerably up-regulated (Figure 5b,d,f).

## 4. Discussion

### 4.1. Cellular Homeostasis and Phagocytic Index of Phagocytes Are Key Factors Determining Whether Sea Urchins Are Resistant to Black Mouth Disease

One of the major functions of the immune system of the sea urchin is phagocytosis. Its main cells mediating immune responses are phagocytes, which search for, trap, and destroy foreign particles [17]. Therefore, in theory, the number of phagocytic cells, the effective cell number (to eliminate cells in states such as necrotic), and phagocytic activity (including phagocytic rate and phagocytic index) in the coelomic fluid of the sea urchins can reflect the immune status of the sea urchin to some extent. Zhao et al. [29] found that lipopolysaccharide stimulation can cause the number of phagocytes to rise in *S. intermedius*. The number of coelomocytes may also be affected by physicochemical parameters of the water such as water temperature, but our experiment was conducted under the same water condition, so the interference of water physicochemical parameters can be excluded. Using pathogen infection, this study similarly observed a rise in phagocyte numbers in sea urchins, suggesting that the enhancement of phagocytosis by increasing the number of phagocytes is one way to enhance immunity in sea urchins. However, this enhancement cannot be accounted for by increased numbers alone considering all three black mouth disease-resistant sea urchins showed less increase in phagocyte numbers than the susceptible sea urchins (*S. intermedius* ♀ × *H. crassispina* ♂). The number of phagocytes does not determine the phagocytosis of the whole organism for pathogenic bacteria. An opposite trend of change has even been reported in other aquatic animals. As demonstrated by Monari et al. [30] in their study of *Chamelea gallina* blood cell function under elevated temperatures, although the number of blood cells increased, the phagocytic index and phagocytic rate decreased. Additionally, Yu et al. [31] found that at a temperature of 30 °C, the total number of blood cells from *Mactra veneriformis* significantly increased, but the phagocytosis decreased.

Compared to phagocytes, the density of the other three types of cells shows relatively small changes. Especially for susceptible sea urchins, the density of these three types of cells did not show any significant changes after infection, while both the densities of colorless spherical cells and red spherical cells in disease-resistant sea urchin *M. nudus* increased significantly 6 h post-infection. Both densities of vibratile cells in disease-resistant sea urchins *H. crassispina* and *H. crassispina* ♀ × *S. intermedius* ♂ significantly increased 3 h post-infection. Studies have shown that colorless spherical cells, red spherical cells, and vibratile cells play a synergistic role in the phagocytic process in sea urchins, mainly providing a supplementary function for phagocytic cells facing bacterial invasion [32,33,34,35]. Therefore, the increase in the density of these three types of cells in disease-resistant sea urchins might help improve their phagocytosis, while in susceptible sea urchins, these cells might not provide significant assistance.

Before pathogen infection, that is, under normal conditions, the necrosis rate and apoptosis rate of phagocytes were approximately 20%, while after pathogen infection, the apoptosis rate and necrosis rate were markedly up-regulated. Apoptosis is an active death process of cells to maintain the stability of the internal environment [30]. The apoptosis rate of black mouth disease-resistant sea urchins was significantly higher than that of susceptible sea urchins 6 h after infection. The black mouth disease-resistant sea urchins have a higher ability to maintain stability by apoptosis. Cell necrosis is the death of cells under the action of pathogenic factors belonging to passive necrosis [36]. At 6 h after infection, the cell necrosis rate of the black mouth disease-resistant sea urchins was significantly higher than that of the susceptible sea urchins. However, this could return to normal in 48 h. The cell necrosis rate of the susceptible sea urchins, however, consistently increased, and this undoubtedly led to the weakening of the overall phagocytosis of the body. It may be deduced from the above that changes in the cell state are one of the key factors in determining illness in a sea urchin. When the susceptible sea urchin is infected by the pathogen, it cannot effectively maintain the body’s homeostasis through apoptosis. With the aggravation of infection, a large number of phagocytes become necrotic, and the sea urchin gradually loses its immunity.

Phagocytic activity represents the ability of cells to phagocytize pathogens or foreign bodies. It, therefore, also represents the immune level of the body. It was found that the phagocytic index of phagocytes of the three black mouth disease-resistant sea urchins was about twice that of the two susceptible sea urchins (*p* < 0.05), indicating that the phagocytic activity of the black mouth disease-resistant sea urchins was stronger than that of the susceptible sea urchins. Although the phagocytic rate of the black mouth disease-resistant sea urchins was higher than that of the susceptible sea urchins, it did not reach a significant level. It may therefore be considered that the black mouth disease-resistant sea urchins primarily obtain disease resistance by phagocytizing more pathogens per phagocyte, i.e., the phagocytic index may better represent the disease resistance of sea urchins than the phagocytic rate.

### 4.2. The Physiological Parameters Related to Phagocytosis of Disease-Resistant Sea Urchins Increase More Significantly for Rapid Sterilization and Can Timely Return Homeostasis

Since massive necrosis of phagocytes occurs, the parameters of ACP, ROS, and T-AOC were measured in this study and then calculated as the mean contribution value (MCV) of single effective phagocytes, an index that eliminates the interference of necrotic cells and more accurately reflects the immune status of sea urchins [16]. Acid phosphatase is the signature enzyme of phagocytic lysosomes. The phagocytosis and encapsulation reactions performed by immune cells are accompanied by the release of acid phosphatase. This can degrade foreign bodies with phosphate esters on the surface by hydrolysis in an acidic environment [37]. Reactive oxygen is the product of the respiratory burst in the phagocytosis process, which has a strong bactericidal function. When there is an excess of reactive oxygen species, it is likely to cause some damage to the body. The antioxidant capacity of the body will then be enhanced to scavenge the excess reactive oxygen radicals to keep the body in a normal state. In this experiment, MCV of ACP, ROS, and T-AOC of the three black mouth disease-resistant sea urchins demonstrated a consistent increase and then decreasing trend after the pathogenic bacterial infection, which was also found in *Chlamys farreri* [38] and *Apostichopus japonicus* [39] after bacterial infection. The up-regulation was considerably higher than that of the two susceptible sea urchins, which facilitated their more rapid killing of the pathogen, while at the later stage of infection (48 h), MCV of ACP, ROS, and T-AOC in the phagocytes of the black mouth disease-resistant sea urchins had returned to normal. The susceptible sea urchins were, however, still in an up-regulated state, indicating that the black mouth disease-resistant sea urchins sterilized quickly and cleared the pathogenic bacteria within 48 h, while the susceptible sea urchins did not remove the pathogenic bacteria completely until 48 h post-infection. The above results indicate that the ability to significantly increase the rapid killing of pathogens by the three physiological parameters related to phagocytosis and timely return of homeostasis is another important factor for the disease resistance of sea urchins.

### 4.3. Similarities and Differences between Hybrid Sea Urchins and Their Parents

The parents used for distant crosses are often required to have complementary advantages in the same trait, and the hybrid progeny are expected to combine the advantages of the parents in that trait. In the present experiment, by comparing the black mouth disease-resistant sea urchins *H. crassispina* with the susceptible sea urchins *S. intermedius* and their crosses, it was found that the vast majority of immune indices related to phagocytosis in the crosses were not considerably different from the maternal parent, while they were markedly different from the paternal parent. Our previous study [40] also found that the heat resistance of the progeny of the cross between *S. intermedius* and *Hemicentrotus pulcherrimus* was closer to their mothers. Cai et al. [41] determined non-specific immune indices such as phagocytosis in *Oreochromis niloticus* and *Oreochromis aurea* and their hybrid progeny and found that the hybrids had stronger maternal bias. Lv et al. [42] found that hybrid scallops had an obvious maternal bias in non-specific immunity by measuring various non-specific immunity indices in the hybrid progeny of *C. farreri* and *Patirtopecten yessoensis*. These results indicate that the crosses have a significant maternal bias in the phagocytosis-related immune activity, i.e., they are close to the maternal parent in disease resistance, which is consistent with the maternal bias of the crosses in external traits. The disease resistance of hybrid sea urchins with black mouth disease-resistant sea urchins as the maternal parent may therefore be related to their strong phagocytosis in production practice.

### 4.4. Screening of Sea Urchin Disease-Resistance Indicators

Black mouth disease has occurred almost every year in sea urchin culture in recent years, and mass mortality has occurred from time to time [8,9,16]. For disease prevention and control, there is an urgent need to find indicators that represent the immunity or disease resistance of sea urchins to predict the occurrence of disease or to screen for parents with high disease resistance. In our previous study [16], it was found that the apoptosis rate of phagocytes, the MCV of ACP, ROS, and T-AOC may be candidates to represent the immunity of sea urchins. However, it is not clear how these indicators can represent the disease resistance in sea urchins. In this study, the typical characteristics of the black mouth disease sea urchins were found by comparing the differences between the resistant and susceptible sea urchins. Phagocytosis was greatly enhanced in the early stage of infection, and after killing the disease bacteria in a short period, phagocytosis was timely returned to normal levels. Based on these findings, two key detection time points may be set, i.e., 3–6 h in the early stage of infection and 48 h in the late stage of infection. The criteria for sea urchin resistance to black mouth disease may be additionally set, i.e., at the early stage of infection, the apoptosis rate of phagocytes, the phagocytic index, the MCV of ACP, ROS, T-AOC were highly up-regulated. At the late stage of infection, the cell necrosis, MCV of ACP, ROS, T-AOC decreased to normal levels. These indicators may be measured by simply extracting the coelomic fluid of sea urchins, which has the advantages of being low damage, rapid, economical, and accurate, and is expected to be applied for the prevention of diseases in sea urchins.

## 5. Conclusions

In conclusion, the findings show that the apoptosis and necrosis rate of phagocytes, phagocytic index, ACP, ROS and T-AOC could indicate disease resistance in sea urchins. These immune indicators of the disease-resistant sea urchins were considerably up-regulated at 3 h to 6 h post-infection and timely down-regulated at 48 h post-infection. Immune indicators may be used for the selection of disease-resistant sea urchins. Our findings might provide important references for disease-resistant breeding of sea urchins.

## Figures and Tables

**Table 1:** Effect of species, time and species–time interaction on immune parameters.

Immune Parameters	Statistical Parameters	Factors		
		Time	Species	Time × Species
Density of phagocytic amoebocyte	F (df)	F (3) = 30.069	F (4) = 25.070	F (12) = 6.406
	*p* value	*p* < 0.0001	*p* < 0.0001	*p* < 0.0001
Density of colorless spherule cell	F (df)	F (3) = 4.230	F (4) = 15.611	F (12) = 5.878
	*p* value	*p* = 0.011	*p* < 0.0001	*p* < 0.0001
Density of vibratile cell	F (df)	F (3) = 4.780	F (4) = 3.879	F (12) = 4.148
	*p* value	*p* = 0.006	*p* = 0.009	*p* < 0.0001
Density of red spherule cell	F (df)	F (3) = 6.835	F (4) =18.544	F (12) = 4.283
	*p* value	*p* = 0.001	*p* < 0.0001	*p* < 0.0001
Apoptosis rate	F (df)	F (3) = 1661.179	F (4) = 12.292	F (12) = 5.279
	*p* value	*p* < 0.0001	*p* < 0.0001	*p* < 0.0001
Necrosis rate	F (df)	F (3) = 230.247	F (4) = 18.252	F (12) = 57.740
	*p* value	*p* < 0.0001	*p* < 0.0001	*p* < 0.0001
phagocytic rate	F (df)	F (2) = 69.699	F (4) = 4.762	F (8) = 1.126
	*p* value	*p* < 0.0001	*p* = 0.004	*p* = 0.375
phagocytic index	F (df)	F (2) = 24.512	F (4) = 7.990	F (8) = 2.887
	*p* value	*p* < 0.0001	*p* < 0.0001	*p* < 0.016
ACP	F (df)	F (3) = 212.771	F (4) = 29.462	F (12) = 35.625
	*p* value	*p* < 0.0001	*p* < 0.0001	*p* < 0.0001
ROS	F (df)	F (3) = 125.294	F (4) = 25.846	F (12) = 20.015
	*p* value	*p* < 0.0001	*p* < 0.0001	*p* < 0.0001
T-AOC	F (df)	F (3) = 132.514	F (4) = 41.273	F (12) = 33.100
	*p* value	*p* < 0.0001	*p* < 0.0001	*p* < 0.0001

## Data Availability

The original contributions presented in the study are included in the article, further inquiries can be directed to the corresponding authors.
